# Organ-Specific Alterations in Fatty Acid De Novo Synthesis and Desaturation in a Rat Model of Programmed Obesity

**DOI:** 10.1186/1476-511X-10-72

**Published:** 2011-05-11

**Authors:** Jennifer K Yee, Wai-Nang P Lee, Guang Han, Michael G Ross, Mina Desai

**Affiliations:** 1 Department of Pediatrics, Los Angeles Biomedical Research Institute at Harbor-UCLA Medical Center, 1124 West Carson Street, Bldg RB-1, Harbor Box 446, Torrance, CA 90502, USA; 2 Department of Obstetrics and Gynecology, Los Angeles Biomedical Research Institute at Harbor-UCLA Medical Center, 1124 West Carson Street, Bldg RB-1, Harbor Box 446, Torrance, CA 90502, USA

## Abstract

**Background:**

Small for gestational age (SGA) leads to increased risk of adult obesity and metabolic syndrome. Offspring exposed to 50% maternal food restriction *in utero *are born smaller than Controls (FR), catch-up in growth by the end of the nursing period, and become obese adults. The objective of the study was to determine stearoyl-CoA desaturase activity (SCD1) and rates of de novo fatty acid synthesis in young FR and Control offspring tissues at the end of the nursing period, as possible contributors to catch-up growth.

**Methods:**

From gestational day 10 to term, dams fed ad libitum (Control) or were 50% food-restricted to produce small FR pups. Control dams nursed all pups. At postnatal day 1 (p1) and p21, offspring body tissues were analyzed by GC/MS, and desaturation indices of palmitoleate/palmitate and oleate/stearate were calculated. SCD1 gene expression was determined by real-time PCR on adipose and liver. Offspring were enriched with deuterium that was given to dams in drinking water during lactation and de novo synthesis of offspring body tissues was determined at p21. Primary adipocyte cell cultures were established at p21 and exposed to U^13^C-glucose.

**Results:**

FR offspring exhibited higher desaturation index in p1 and p21 adipose tissue, but decreased desaturation index in liver at p21. SCD1 gene expression at p21 was correspondingly increased in adipose and decreased in liver. FR subcutaneous fat demonstrated increased de novo synthesis at p21. Primary cell cultures exhibited increased de novo synthesis in FR.

**Conclusions:**

Adipose tissue is the first site to exhibit increased de novo synthesis and desaturase activity in FR. Therefore, abnormal lipogenesis is already present prior to onset of obesity during the period of catch-up growth. These abnormalities may contribute to future obesity development.

## Background

Obesity is one of the most serious public health problems of the century. Currently, 65% of adults in the United States are overweight, with 33% of adults [1] and 17% of children obese [2]. Obesity and its related diseases are a leading cause of death in western society, as obesity increases risk for hypertension, coronary heart disease, stroke, and diabetes. As childhood obesity is a major risk factor for adult obesity, the 17% incidence of childhood obesity portends a further increase in the prevalence of adult obesity.

The Barker hypothesis of gestational programming is supported by human epidemiologic and animal studies which have convincingly demonstrated that low birth weight and small for gestational age (SGA) newborns have increased risk of adult obesity and metabolic syndrome [3-5]. Animal models of programmed obesity produce SGA newborns who catch-up in growth by the end of the nursing period, then develop adult obesity. The mechanism(s) for programmed obesity in these animal models have been attributed to hyperphagia, enhanced adipogenesis, hyperinsulinemia, and hyperleptinemia [6,7]. The programmed offspring exhibit a phenotype of increased adipogenesis and adipocyte differentiation and a propensity for fat storage.

Synthesis of fatty acids (via de novo lipogenesis in liver and adipose) and triglycerides are important factors in fat accumulation. While obesity and metabolic syndrome involve abnormalities in the liver, adipose tissue and muscle, the organ-specific changes in lipogenesis have not yet been studied. Triglycerides destined for fat storage in adipose tissue are composed of fatty acids from dietary sources and from de novo synthesis. De novo synthesized fatty acids can undergo modification through creation of double bonds via desaturation, and/or further lengthening via chain elongation, thereby implicating dysregulation of long chain fatty acid metabolism in the mechanism of obesity.

Adipose tissue functions in lipid homeostasis to maintain energy balance through activities of the fatty acid metabolic network. While de novo synthesis and chain elongation promote energy storage, breakdown of fatty acids by chain shortening and β-oxidation promote energy release. Perturbation of the metabolic network may shift the energy balance toward increased energy release, or, as in obesity, increased energy storage. Metabolite profiling of fatty acids can therefore provide insights into abnormalities of lipogenesis. Notably, in obese humans and animal models, stearoyl-CoA desaturase enzyme 1 (SCD1), which converts saturated palmitate (16:0), and stearate (18:0) to their monounsaturated products palmitoleate (16:1n-7) and oleate (18:1n-9), respectively, is upregulated, and thus increases the monounsaturated to saturated fatty acid ratio [7,8]. Monounsaturated fatty acids, such as palmitoleate and vaccenate (18:1n-7, made from elongation of palmitoleate), but especially oleate, are preferred substrates for triglyceride synthesis. Since triglycerides become incorporated into adipose tissue for storage, an increase in the monounsaturated to saturated fatty acid ratio, therefore, increases propensity for fat storage.

Use of stable isotopes in profiling enables determination of de novo synthesis rates within a specified time period in metabolically active tissues. Stable isotopes frequently used in vitro and in vivo for study of fatty acid metabolism include deuterium-enriched water (D_2_0) [9,10], and ^13^C-enriched glucose or fatty acids [11]. The ^2^H or ^13^C is incorporated during fatty acid synthesis and therefore provides dynamic information on metabolic activities that took place within the experimental period.

We have established a rat model for nutritional programming of adult obesity [6,12]. Our studies show that offspring exposed to 50% maternal food restriction (FR) *in utero *are SGA at birth. When nursed by normally fed control dams, they exhibit rapid catch-up growth by 3 weeks of age. They subsequently develop adult obesity with increased percent body fat and insulin resistance. Importantly, the FR offspring exhibit increased expression of adipogenic genes and hypertrophic adipocytes prior to onset of obesity [13] implicating increased adipogenesis as a likely source of these abnormalities. Whether changes in lipogenesis contribute to adipose tissue development in FR is unclear.

Adipose, liver, and muscle can all exhibit dysregulated lipogenesis in obesity. Nonetheless, the dynamics of tissue-specific lipogenesis during the catch-up growth phase have not been previously studied. We hypothesized that enhanced lipogenesis during catch-up growth contributes to programmed obesity. We utilized a metabolomic approach to investigate pathways of fatty acid synthesis in the same rat model that has been previously well-described by our group [6,12-14]. We thus determined SCD1 activity and rates of fatty acid de novo synthesis during the nursing period in major metabolic tissues (adipose tissue, liver, muscle) and plasma prior to onset of obesity in FR male offspring. Additionally, we hypothesized that increased de novo synthesis in adipose tissue is an intrinsic cellular response. Therefore, we established adipose primary cell cultures from offspring to determine if increased de novo synthesis would be enhanced under the cell culture system.

## Methods

### Animal studies

Studies received approval from the Animal Care Committee at the Los Angeles Biomedical Research Institute at Harbor-UCLA and were in accordance with the American Accreditation Association of Laboratory Care, and conforming to the Public Health Service Policy on Humane Care and Use of Laboratory Animals.

A description of the maternal food restriction (FR) rat model has been previously published [6]. In brief, first-time pregnant Sprague-Dawley rats (Charles River Laboratories, Hollister, CA) were housed in an animal facility that had controlled 12/12 hour light/dark cycles, and were provided with constant temperature and humidity conditions. On day 10 of gestation (e10), dams were given either a standard laboratory ad libitum chow diet (Lab Diet 5001, Brentwood, Missouri), or were 50% food-restricted to produce SGA pups. Dams continued the assigned diets during pregnancy and lactation. After birth, on day 1 (p1), individual pups were weighed and median weight obtained. To standardize nursing, litters were culled to 8 pups per dam (4 males and 4 females) to include the offspring of median weights. FR offspring were cross-fostered to ad libitum-fed control dams (also known as Cross-over dams). To control for cross-fostering technique, the control pups were also cross-fostered to Control dams.

### Blood and Tissue Collection

1 day old pups were decapitated and plasma samples were pooled per litter (males and females) and subcutaneous fat (SC fat), liver, and muscle were collected from male offspring only and snap-frozen in liquid nitrogen for analysis. In all cases except liver, 6 litters per group were studied. Livers from 6 pups in each group were individually studied. Note that retroperitoneal (RP) fat was not collected due to limited availability of this tissue at this age.

At 3 weeks of age, male offspring (n = 5 Control, n = 6 FR) were weighed, anesthetized by 5% isoflurane/2% oxygen by mask and blood collected via cardiac puncture. Following this, animals were euthanized by an overdose of pentobarbital (200 mg/kg i.p.) and tissues (SC and RP fat, liver, and muscle) were collected and snap-frozen in liquid nitrogen for analysis.

This manuscript describes studies on males only. Gender differences in development of obesity exist in this model so females were studied separately and will be reported separately.

### Deuterium Studies in Nursing Pups

Lactating dams nursing Control and FR pups (n = 8 each group) received deuterium as a stable isotope tracer. On postnatal day 7 (p7), dams received a 2% (of body weight) intraperitoneal injection of 99.9% deuterium with 0.9% sodium chloride (normal saline). Then, from p7 to p21, dams were provided 6% deuterium-enriched drinking water. The concentration of deuterium used was to maintain an approximate 3% body water enrichment of deuterium. The deuterium enrichment in offspring occurred through nursing and perhaps a small contribution directly from drinking deuterated water toward the end of the nursing period. Blood was collected by tail bleeds on p12, p15, and p21 from lactating dams which corresponded to days 5, 8, and 14 of deuterium enrichment. At p21, male offspring were sacrificed and blood and tissues (SC fat, RP fat, liver, and muscle) were collected as stated above

### Primary adipocyte cell cultures

Primary adipocyte cell cultures on RP fat were studied to evaluate if differences between Control and FR could be preserved, or enhanced in a cell culture system. At p21, RP fat was collected from Control and FR males (n = 8 per group). Pooled adipose tissue was minced, digested with collagenase type II (5000 U/g) in Krebs-Ringer solution for 30 min at 37°C, filtered through 200 μm mesh nylon filter and centrifuged at 500 × g for 5 min. The cells were resuspended in high glucose (450 mg/dl) DMEM (Invitrogen Carlsbad, CA) with 10% FBS and 1% Antibiotic-Antimycotic (Invitrogen Carlsbad, CA), seeded into flasks and incubated at 37°C and 5% CO2. Following 100% confluence, adipocyte differentiation was induced with dexamethasone (1 μM), methylisobutylxanthine (0.1 mM), and insulin (10 μg/ml) for 10 days. After differentiation, cells were incubated with high glucose DMEM medium containing 50% [U^13^C]-glucose. After 24 hours, cells were harvested and frozen at -80°C for analysis.

### Fatty acid extractions

Total fatty acids (phospholipids, triglycerides, cholesteryl esters and free fatty acids) were extracted from plasma, tissues, and cells from adipocyte cultures, using a method by Lowenstein et al [15]. Briefly, tissue (~50 mg liver or muscle; ~30 mg adipose tissue) or plasma samples (50 μl), or adipocytes were saponified in 200 proof ethanol and 30% KOH (w/v) in a 1:1 volume overnight on a 70°C heating block. Samples were acidified with HCl, fatty acids were extracted three times with petroleum ether and air dried. Fatty acids were derivatized as methyl esters using 0.5 N methanolic HCl, dried under a nitrogen stream and subsequently reconstituted with hexane for gas chromatography/mass spectrometry (GC/MS) analysis.

For fatty acid quantification, SC fat was cut and weighed, then saponified with a known quantity of heptadecanoic acid-d3 (tri-deuterated) as an internal standard.

### Gas chromatography/mass spectrometry

GC/MS analysis was carried out using a Hewlett-Packard model 5973 selective mass detector connected to a model 6890 gas chromatograph. Fatty acids were analyzed as their methyl esters after derivatization. Palmitate, palmitoleate, stearate, oleate, and vaccenate were separated on the gas chromatograph with a Bpx70 column (30-m length, 250- μm diameter, 0.25- μm film thickness) from SGE, Inc. (Austin, TX). The GC conditions were as follows: helium flow rate, 1 ml/min; initial oven temperature, 150°C, which was programmed to increase at 3°C/min to a final temperature of 221°C. The expected retention times for palmitate, palmitoleate, stearate, oleate, and vaccenate under these conditions were as follows: 6.5, 7.1, 9.4, 10.0, and 10.1 min, respectively. Mass spectra of fatty acids were acquired using electron impact ionization and selective ion monitoring. Ion clusters monitored for the quantitation of isotopomers were mass-to-charge ratio (m/z) 270 for palmitate, m/z 298 for stearate, m/z 264 for oleate. Distribution of the mass isotopomer was determined from the spectral data using a method previously described by Lee et al [16] that corrects for the contribution of derivatizing agent and ^13^C natural abundance to the mass isotopomer distribution of the compound of interest. Each compound of interest includes the sum of isotopomer peaks within a cluster. The resulting mass isotopomer distribution was expressed in molar fractions (m0, m1, m2, m3, etc.) corresponding to the fraction of molecules that contain 0, 1, 2, 3, ...deuterium substitutions.

### Fatty acid profile

The distribution of the fatty acids of interest observed by gas chromatography are presented graphically. By convention, each fatty acid is expressed as a relative percentage normalized to palmitate in the same tissue, with palmitate set at 100%.

### Desaturation Index

The desaturation index is the ratio of monounsaturated fatty acid to corresponding saturated fatty acid determined by the integrated areas under the gas chromatogram peaks. The ratios of palmitoleate/palmitate (16:1/16:0), oleate/stearate (18:1n-9/18:0), and vaccenate/stearate (18:1 n-7/18:0) were determined for this study^a^.

### Stearate/palmitate ratio

Stearate can be made directly from de novo synthesis or from chain elongation of palmitate. The stearate/palmitate ratio was determined to represent production and disappearance of stearate relative to production and disappearance of palmitate.

### Calculation of de novo synthesis and acetyl-CoA enrichment in primary adipocyte cell cultures using U^13^C-glucose

Precursor acetyl-CoA enrichment was calculated from the consecutive mass isotopomer ratio M+4/M+2 of palmitate and M+6/M+4 of stearate, from incorporation of [1,2-^13^C] acetate produced from U^13^C-glucose in the medium. The fraction of new synthesis was calculated using mass isotopomer distribution analysis (MIDA) [11], as previously described. These calculations used information from the "light" fraction of molecules with peaks corresponding to m_2_, m_4_, and m_6 _molecules from incorporation of [1,2-^13^C]acetyl-CoA in de novo synthesis of palmitate and stearate [16,17].

### Calculation of de novo synthesis in deuterium studies

The fraction of new synthesis (FNS) representing de novo synthesis was calculated using previously described methods [18,19]. Briefly, FNS = ME(fatty acid)/(p × N), in which ME (molar enrichment) is the average number of deuterium atoms incorporated per molecule calculated from mass isotopomer distribution, p is the deuterium enrichment (specific activity) in water which is determined using the m2/m1 consecutive mass isotopomer ratio, and N is the number of deuterium per molecules incorporated from body water per molecule of fatty acid (e.g. 21 for palmitate).

For quantification of fatty acids made over the 14 day period, the amounts of palmitate, stearate, and oleate per mg of SC fat were first determined based on GC peak areas relative to the heptadecanoic acid-d3 internal standard, and then calculations were made using the FNS. The amounts of fatty acids made are expressed as the average amounts made per mg of fat tissue per day.

### SCD1 mRNA expression by real-time PCR

RNA was extracted from adipocytes using the Qiagen RNeasy Lipid Tissue Mini Kit, then DNase-treated (Ambion Turbo DNA-free kit). RNA concentration was determined on the Nanodrop 2000 spectrophotometer (Thermo Scientific). cDNA was made with 2 μg of RNA using the Invitrogen Superscript III First Strand Synthesis System for RT-PCR. SCD1 primers were generated from NCBI reference sequence [NCBI: NM 139192.2] by Primer Express software (Applied Biosystems). SCD1 forward primer sequence 5'→3': CAGAGCCAGGTGCCACTTTT; SCD1 reverse primer sequence 5'→3': TGCTAGAGGGTGTACCAAGCTTT. 18S was used as the reference gene. 18S forward primer sequence 5'→3'CTTAGAGGGACAAGTGGCG; 18S reverse primer sequence 5'→3'GGACATCTAAGGGCATCACA. Real-time PCR was performed using SYBR Green reporter dye on the ABI Prism 7000 sequence detection system with annealing temperature of 60°C, along with 18S as the reference gene. Results were converted to ΔΔCt values, and expression of SCD1 was normalized to 18S levels. SCD1 expression in FR is presented as fold change relative to Controls.

### Statistical Analysis

Data are presented as the mean, ± SE. Comparisons are made between Control and the FR group using unpaired t-test.

## Results

### Metabolic Phenotype - In Vivo

#### Rat Offspring Weights

There was no difference between Control and FR litter sizes at birth. As previously published, FR offspring were smaller than Controls at p1 (5.2 ± 0.2 vs 7.0 ± 0.3 gm) [6]. When nursed normally, FR offspring body weights were similar to the controls at p21 (FR 42.2 ± 2.0 vs Controls 48.5 ± 3.3 gm).

#### Dam Weights

No differences between Control and Cross-over dam weights were present during lactation. At p1, Control and Cross-over weights were, respectively, 297.6 ± 5.3 and 298.1 ± 6.6 gms, and both groups gained weight during early lactation. At p14, Control and Cross-over dam weights were 340.3 ± 6.2 and 351.2 ± 6.8 gms. At p20, the weights had decreased to 334.0 ± 5.7 and 345.1 ± 7.8 gms, respectively. Upon sacrifice at p21, no visible milk globules were readily available for collection from the mammary tissue.

#### Fatty acid profile

In general, gas chromatography peaks were observed for the following saturated fatty acids: (myristate, 14:0; palmitate, 16:0; stearate, 18:0) and monounsaturated (palmitoleate 16:1n-7; oleate, 18:1n-9; vaccenate, 18:1n-7). The fatty acid distribution exhibited tissue and age specificity as described below.

##### Dams

**Figure 1:**
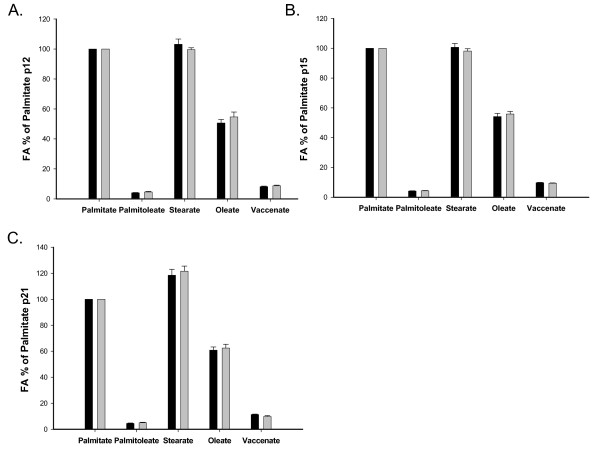
**Fatty acid distribution represented as percent of palmitate in dam plasma on p12, p15, and p21**. Black bars represent the Control dams, and gray bars the Cross-Over dams. Stearate is similarly prominent as palmitate in the dams until p21, when stearate becomes the most prominent fatty acid.

The plasma fatty acid profile was comparable in both groups of dams. At p12 and p15 palmitate and stearate levels were similarly high though at p21, stearate was the most prominent fatty acid (Figure 1).

##### Offspring

**Figure 2:**
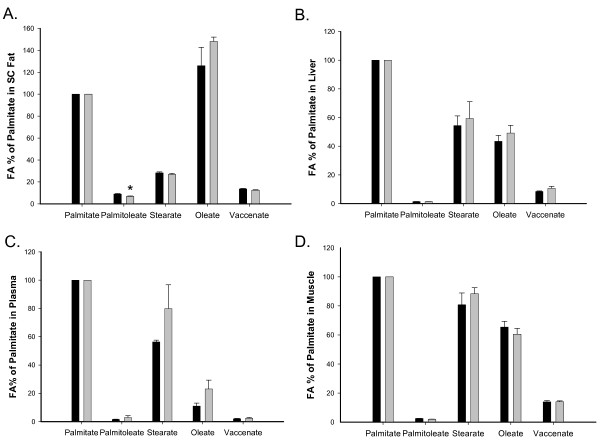
**Fatty acid distribution in p1 body tissues, expressed as percent of fatty acids normalized to palmitate, with palmitate set at 100%**. Black bars represent the Control group, and gray bars the FR group. *p < 0.05 when comparing the two groups. Tissues were from p1 males except for plasma which was pooled males and females. In SC fat (A), oleate was the most prominent fatty acid in both Control and FR groups. In SC Fat, percent of palmitoleate was decreased in FR versus Controls. In liver (B), plasma (C), and muscle (D), palmitate was the most prominent fatty acid, followed by stearate, and then oleate.

At p1 (Figure 2), in both FR and Controls, oleate was the most abundant fatty acid in SC fat, whereas palmitate was the most abundant in liver, muscle and plasma. Comparison between FR and Controls at p1 revealed a significant decrease in SC fat palmitoleate.

**Figure 3:**
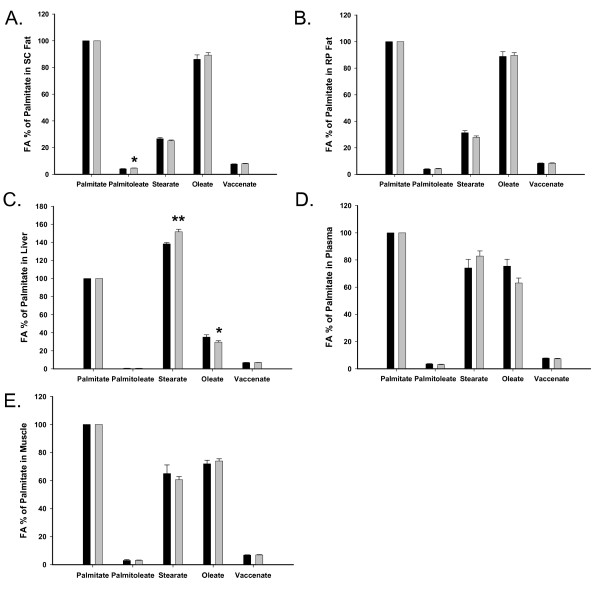
**Fatty acid distribution at p21 in various tissues**. The percent of fatty acid is normalized to that of palmitate. Black bars represent the Control group, and gray bars the FR group. *p < 0.05 and **p < 0.01 when comparing the two groups. The fatty acid distribution was similar between the SC fat (A) and RP fat (B). In liver, stearate was increased but oleate was decreased in FR compared to Controls. The fatty acid distribution of plasma (D) and muscle (E) were similar. The trend toward differences between FR and Control stearate and oleate in plasma (D) appeared to reflect the differences in liver (C).

At p21 (Figure 3), in both FR and Controls, oleate and palmitate were the most prominent fatty acids in SC and RP fat. In liver, stearate was the most prominent fatty acid, while in muscle and plasma, palmitate continued to be more prominent. Comparison of FR and Controls at p21 revealed that FR exhibited increased palmitoleate in SC fat, decreased oleate in liver, but increased stearate in liver.

#### Desaturation indices

Desaturation indices are expressed in arbitrary units, ± standard error of the mean.

##### Dams

At p12, there were no other differences in the desaturation indicies other than a slight increase in the oleate/stearate ratio in Cross-Over dams at p12 (Table 1).

**Table 1:** The desaturation indices and the stearate/palmitate ratio for rat dam plasma on p12, p15, and p21.

	Palmitoleate/Palmitate Ratio		Oleate/Stearate Ratio		Vaccenate/Stearate Ratio		Stearate/Palmitate Ratio	
**Day**	**Control**	**Cross-Over**	**Control**	**Cross-Over**	**Control**	**Cross-Over**	**Control**	**Cross-Over**

p12	0.040 ± 0.002	0.046 ± 0.004	0.48 ± 0.02	0.55 ± 0.03*	0.080 ± 0.004	0.088 ± 0.004	1.03 ± 0.04	1.00 ± 0.01

p15	0.042 ± 0.001	0.043 ± 0.002	0.54 ± 0.03	0.57 ± 0.02	0.096 ± 0.002	0.096 ± 0.003	0.98 ± 0.03	1.03 ± 0.02

p21	0.045 ± 0.004	0.050 ± 0.004	0.52 ± 0.05	0.53 ± 0.03	0.095 ± 0.006	0.082 ± 0.005	1.18 ± 0.05	1.22 ± 0.04

*Mean p < 0.05 in comparison to Control group

##### Offspring

At p1, SC fat and plasma both exhibited increased oleate/stearate ratios in FR (Table 2). Interestingly, SC fat palmitoleate/palmitate ratio was significantly decreased in FR. No differences were seen in liver and muscle.

**Table 2:** The desaturation indices and the stearate/palmitate ratio at p1

	Palmitoleate/Palmitate Ratio		Oleate/Stearate Ratio		Vaccenate/Stearate Ratio		Stearate/Palmitate Ratio	
**Body Tissue**	**Control**	**FR**	**Control**	**FR**	**Control**	**FR**	**Control**	**FR**

SC Fat	0.09 ± 0.003	0.07 ± 0.003*	4.94 ± 0.22	5.53 ± 0.09*	0.48 ± 0.02	0.47 ± 0.02	0.28 ± 0.01	0.26 ± 0.02

Liver	0.009 ± 0.001	0.016 ± 0.0005	0.75 ± 0.1	0.95 ± 0.2	0.16 ± 0.02	0.19 ± 0.03	0.57 ± 0.06	0.59 ± 0.1

Plasma*^b^*	0.02 ± 0.002	0.03 ± 0.01	0.20 ± 0.04	0.37 ± 0.06*	0.07 ± 0.03	0.04 ± 0.01	0.56 ± 0.01	0.70 ± 0.10

Muscle	0.031 ± 0.004	0.026 ± 0.001	0.85 ± 0.12	0.70 ± 0.07	0.14 ± 0.03	0.16 ± 0.01	0.81 ± 0.08	0.88 ± 0.04

*p < 0.05 in comparison to Control group

*^b^*SC fat, liver, and muscle are from males, and plasma is pooled from males and females due to small volumes in these animals at this age.

At p21 (Table 3), both SC and RP fat in FR exhibited changes consistent with obesity; there was a marked increase in all three desaturation indices (palmitoleate/palmitate, oleate/stearate, vaccenate/stearate). In contrast, FR liver showed decreased ratios of oleate/stearate and vaccenate/stearate. No differences were evident either in plasma or muscle desaturation indices between the two groups.

**Table 3:** The desaturation indices and the stearate/palmitate ratio in p21 offspring tissues.

	Palmitoleate/Palmitate Ratio		Oleate/Stearate Ratio		Vaccenate/Stearate Ratio		Stearate/Palmitate Ratio	
**Body Tissue**	**Control**	**FR**	**Control**	**FR**	**Control**	**FR**	**Control**	**FR**

SC Fat	0.041 ± 0.007	0.046 ± 0.008*	3.23 ± 0.005	3.51 ± 0.001*	0.29 ± 0.004	0.31 ± 0.006*	0.27 ± 0.008	0.25 ± 0.006

RP Fat	0.037 ± 0.003	0.043 ± 0.002^¥^	2.85 ± 0.11	3.19 ± 0.11*	0.27 ± 0.01	0.30 ± 0.01*	0.31 ± 0.02	0.28 ± 0.01

Liver	0.0061 ± 0.0005	0.0059 ± 0.0001	0.25 ± 0.04	0.19 ± 0.02*	0.049 ± 0.003	0.046 ± 0.001	1.38 ± 0.03	1.51 ± 0.07*

Plasma	0.036 ± 0.003	0.031 ± 0.002	1.07 ± 0.15	0.80 ± 0.08	0.11 ± 0.01	0.09 ± 0.01	0.74 ± 0.06	0.80 ± 0.05

Muscle	0.025 ± 0.003	0.029 ± 0.002	1.17 ± 0.17	1.24 ± 0.09	0.16 ± 0.01	0.17 ± 0.01	0.65 ± 0.06	0.61 ± 0.04

* p < 0.05 in comparison to Control group

^¥ ^p = 0.05

#### Stearate/palmitate ratio

##### Dams

There were no differences in the stearate/palmitate ratio (Table 1).

##### Offspring

At p1, the stearate/palmitate ratio was not significantly different between FR and Controls (Table 2). At p21 (Table 3), only FR liver exhibited an increased ratio.

#### SCD1 mRNA expression by real-time PCR

**Figure 4:**
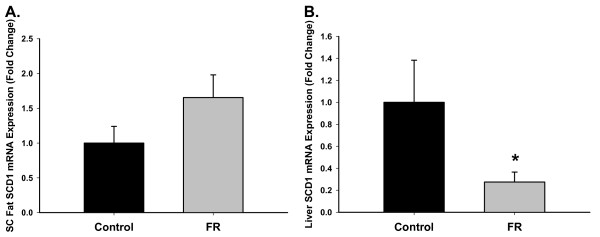
**SCD1 mRNA expression in FR and Control offspring at p21 in A) SC fat and B) liver**. Corresponding to the desaturation indices, SCD1 mRNA expression in FR demonstrates a strong trend toward an increase in SC fat (p = 0.06), and a significant decrease in liver (*p < 0.05).

3 week old FR offspring demonstrated a strong trend to an increase in SCD1 mRNA expression in SC fat (Figure 4), with FR expression 1.65 times that of Controls (A). FR demonstrated a significant decrease in liver, with expression 0.28 times that of Controls (B).

#### De novo fatty acid synthesis

Deuterium enrichment in body water reached a plateau of about 3-4% in the fatty acids of all offspring tissues and dam plasma, with no differences between Control and FR offspring, or Control and Cross-Over dams (data not shown).

##### Dams

**Figure 5:**
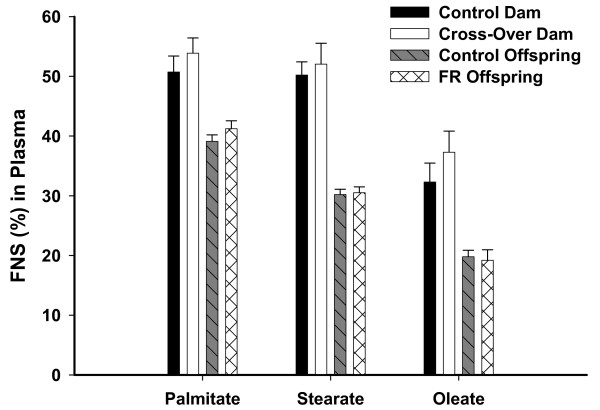
**The FNS of palmitate, stearate, and oleate, for the plasma of dams and offspring at p21**. The FNS of all three fatty acids is much greater in dams than in offspring. There were no differences between Control and Cross-Over dams or Control and FR offspring in plasma.

In both groups (Table 4), the pattern of fatty acid FNS was similar at p12 and p15, with all three fatty acids in similar ranges. However, at p21, the oleate FNS in both groups decreased in abundance compared to palmitate and stearate. There were no differences between Control and Cross-over groups in the FNS of the fatty acids at any time point. Dam plasma fatty acid FNS at p21 were all higher than offspring plasma FNS (Figure 5).

**Table 4:** The fraction of new synthesis (FNS) of palmitate, stearate, and oleate in p21 offspring and dam plasma

		Control			FR or Cross-over		
	**Body Tissue**	**FNS Palmitate (%)**	**FNS Stearate (%)**	**FNS Oleate (%)**	**FNS Palmitate (%)**	**FNS Stearate (%)**	**FNS Oleate (%)**

Dams	Plasma p12	44.1 ± 1.7	40.9 ± 2.1	44.7 ± 1.7	44.9 ± 1.6	46.1 ± 2.0	42.3 ± 1.6
	
	Plasma p15	46.6 ± 1.6	42.9 ± 1.8	52.9 ± 2.1	47.3 ± 1.2	46.0 ± 1.9	50.7 ± 2.1
	
	Plasma p21	50.7 ± 2.7	50.2 ± 2.2	32.3 ± 3.2	53.9 ± 2.6	52.4 ± 3.5	37.3 ± 3.5

Offspring	SC Fat	40.0 ± 1.4	22.5 ± 1.1	17.8 ± 1.0	43.5 ± 1.1*	24.1 ± 1.0	17.6 ± 1.2
	
	RP Fat	42.5 ± 1.3	25.7 ± 0.7	20.5 ± 0.7	44.1 ± 1.7	26.2 ± 1.2	19.3 ± 0.7
	
	Liver	44.9 ± 0.8	35.3 ± 0.8	27.7 ± 0.2	46.1 ± 1.3	36.2 ± 1.2	25.9 ± 0.6
	
	Plasma	39.1 ± 1.1	30.2 ± 0.9	19.8 ± 1.1	41.2 ± 1.3	30.5 ± 1.0	19.2 ± 1.7
	
	Muscle	46.8 ± 0.8	30.1 ± 0.5	29.6 ± 0.5	46.7 ± 1.2	30.5 ± 0.9	27.0 ± 1.2

*p < 0.05 in comparison with Control group

##### Offspring

The pattern of FNS was similar in all tissues with palmitate being the most abundant followed by stearate and oleate. At p21, FNS of palmitate in SC fat was significantly increased in FR compared to Controls (Table 4). There was a strong trend to an increase in palmitate made per mg of SC fat per day in FR offspring (Table 5).

**Table 5:** Average amounts of fatty acids made per mg of SC fat per day

	Average made per mg of tissue per day (μg/mg/day)	
**Fatty Acid**	**Control**	**FR**

Palmitate	3.19 ± 0.22	3.85 ± 0.16^¥^

Stearate	0.36 ± 0.03	0.40 ± 0.06

Oleate	1.02 ± 0.02	1.08 ± 0.03

^¥ ^p = 0.06 in comparison with Control group

### Metabolic Phenotype - Primary Adipocyte Cell Cultures (In vitro)

#### De novo fatty acid synthesis

Although the in vivo study showed no difference in de novo synthesis of fatty acids in RP fat from Control and FR offspring, exposure of adipocytes to high glucose medium in cell culture delineated distinct differences between the groups. FR offspring exhibited increased FNS of palmitate and stearate as Controls, but similar FNS of oleate (Table 6).

**Table 6:** Fraction of new synthesis of fatty acids and acetyl-CoA enrichment of palmitate in primary adipocyte cell cultures

	FNS Palmitate	Acetyl-CoA Enrichment Palmitate	FNS Stearate	FNS Oleate
Control	25.4 ± 0.4	0.118 ± 0.001	7.9 ± 0.2	1.5 ± 0.1

FR	31.0 ± 0.1*	0.133 ± 0.001*	9.2 ± 0.1*	1.7 ± 0.1

*p < 0.05 in comparison with Control group.

#### Acetyl-CoA Enrichment

The acetyl-CoA enrichment represents glucose utilization toward fatty acid synthesis. Consistent with the FNS in vitro, the acetyl-CoA enrichment for palmitate was increased in the FR offspring (Table 6).

## Discussion

The novel approach of metabolic profiling combined with stable isotope use demonstrated increased lipogenesis in adipose tissue during the catch-up growth phase in the maternal food restriction model of programmed obesity. Since the programmed FR offspring continue to grow and surpass Controls to have increased fat mass in adulthood, this increased lipogenesis is a potential contributor to the development of obesity.

The rat dam plasma fatty acid profile and desaturation index may be thought to influence the offspring fatty acid profiles through nursing. The dam plasma measurements were determined at several time points not only to track increasing deuterium enrichment, but also to observe whether increased demand for lactation from hyperphagic FR offspring [14] led to any differences in maternal fatty acid profiles. There was only one timepoint, p12, during which the oleate/stearate ratio was increased in Cross-Over dams, suggesting that FR offspring demand can affect milk fatty acid composition. However, this was an isolated difference, and fatty acid profiles and desaturation indices were similar at p21 and therefore could not account for differences between Control and FR offspring at p21. Whether or not the transiently increased desaturation index correlates to any physiologic changes in the offspring (such as rate of growth at p12) would require further studies.

The dam plasma FNS levels were more prominent than those of the respective offspring fatty acids, which suggests that dam FNS possibly contributes significantly to offspring FNS. However, maternal FNS again does not account for differences between Control and FR offspring. Instead, the dam FNS pattern appears consistent with demand for fatty acids for lactation, with oleate FNS being the most prominent on p15, but decreasing dramatically by p21. The maternal weights in both groups decreased during this time. Therefore, the decreased oleate FNS suggests dilution of the deuterated oleate pool by recycling mobilization from adipose tissue since oleate is a major component of adipose tissue fatty acids. By p21, the dams may be mobilizing its preexisting fat stores in order to keep up with demand for oleate for lactation at this age.

The desaturation index is increased in animal models of obesity [20] and obese humans [8]. The desaturation index has been shown to correlate with SCD1 mRNA expression, protein expression, and enzyme activity [21] and is therefore a good measure of SCD1 activity. We observed differences in the desaturation indices between Controls and FR offspring at p1 and p21. In p1 SC fat, FR exhibited an increased oleate/stearate desaturation index, and decreased palmitoleate/palmitate index. While the opposing directions of the two desaturation pathways appear in conflict with obesity studies on SCD1 activity (increased in obesity), our findings support data that the palmitate-to-palmitoleate desaturation pathway is compartmentalized from the stearate-to-oleate pathway [11,22]. Moreover, palmitoleate has recently been suggested to function as a "lipokine" [23] with anti-inflammatory properties, communicating as a signaling hormone to liver and muscle to improve insulin sensitivity. In this context, the reduced adipose palmitoleate/palmitate desaturation index at p1 may therefore be consistent with increased metabolic risk.

The p21 desaturation pattern and fatty acid profile suggest physiologic changes by p21. At p21, the increased oleate/stearate and vaccenate/stearate desaturation indices in FR SC and RP fat, accompanied by the strong trend to increased SCD1 expression in SC fat, implicate SCD1 activity in the ultimate development of adiposity. In contrast, the liver has a decreased oleate/stearate ratio with a corresponding increased stearate/palmitate ratio, suggesting an accumulation of stearate from lack of desaturation. While it has been shown that starvation decreases hepatic SCD1 gene expression [24], our FR offspring continue to exhibit lower SCD1 activity accompanied by lower SCD1 gene expression during normal nursing, suggesting a programmed effect from the nutrient deprivation. The increased palmitoleate/palmitate ratio in FR SC fat at p21 indicates that SCD1 is under the influence of different factors at p21 versus p1, altering the palmitoleate proportions during these two ages. An important factor is likely the difference in nutrient exposure during these two time points. It has been suggested that palmitoleate produced by fat tissue may provide negative feedback to the liver [25], suppressing SCD1. Our finding of decreased liver oleate/stearate ratio at p21 would appear to be consistent with this hypothesis. However, the biological functions of palmitoleate are still largely unknown and the mechanisms behind the fatty acid metabolic differences seen at p21 versus p1 require further investigation. Whether these differences in tissue desaturation indicies persist in adulthood also requires investigation.

Use of deuterium-enriched water is a useful technique for demonstrating the rate of fatty acid synthesis during a specified time period. Deuterium studies, for example, have enabled determination of de novo synthesis patterns during early life and in response to high-fat feeding in the Zucker rat model of obesity and leptin resistance. Zucker pups have increased de novo lipogenesis in adipose tissue leading to increased triglyceride synthesis during the early suckling period [10], and increased adipocyte hyperplasia after adolescence. As obese adults, the Zucker rat does not suppress its de novo lipogenesis after high fat feeding [26]. For perspective, additional examples of de novo synthesis rates determined after deuterium enrichment in different types of rat tissues are shown in Table 7.

**Table 7:** De novo synthesis rates after varying periods of deuterium enrichment in rat tissues

Source of Data and Species	Tissue	% Deuterium in drinking water	p (% enrichment in plasma water)	Days of enrichment	FNS Palmitate (%)	FNS Stearate (%)
Lee et al, AJP, 1994 [34] Sprague-Dawley young adult	Liver	4%	2.5	7 days	48.53	39.77
			
			2.47	14 days	39.57	31.67
			
			2.76	28 days	43.83	32.42
			
			2.8	56 days	50.17	37.07

Bassilian et al, AJP, 2002 [35] Zucker lean	Plasma triglycerides	6%	Not provided	14 days	70.70	61.03
	
	Epididymal fat	6%	Not provided	14 days	~18*	~12*

Ajie et al, AJP, 1995 [36] HRS/J and Sprague-Dawley	Spinal cord	4%	Not provided	56 days	79.35	65.66

* inferred from graph

In our study, measurement of de novo synthesis over 14 days during the nursing period revealed information on fatty acid turnover. In both Controls and FR, the FNS of palmitate was most prominent, followed by stearate, then oleate, indicating that FNS palmitate is least diluted by preexisting palmitate, but stearate and oleate exhibit more dilution by their preexisting pools. A less abundant FNS oleate in SC and RP fat compared to muscle and liver indicate higher turnover and recycling of preexisting oleate in the fat. Though de novo synthesis (and accumulation of dietary fatty acids) appears active in all tissues at p21, differences among the tissues in fatty acid synthesis and mobilization are evident.

Determination of de novo synthesis during 14 days of the catch-up growth period also revealed an increase in FNS of palmitate at p21 in offspring SC fat. As previously discussed, the dam FNS contribution does not account for the FNS difference in FR offspring. Therefore, SC fat is the first site to exhibit programmed abnormal lipogenesis in this model. The high fat diet of dam's milk (protein 24.6%, fat 69.5%, carbohydrates 5.9%) [27] would be expected to suppress offspring de novo lipogenesis, but it failed to suppress it in the FR group to the same degree as the Control group.

There was a strong trend to an increase in the average amount of palmitate made per mg of SC fat per day in FR compared to Controls. This was averaged from the amount made over a 14 day period, and does not account for day-to-day variation in production. This result is consistent with increased lipogenesis during catch-up growth in FR offspring. However, it does not represent total palmitate made in the fat pads per day, which could not be determined within the scope of the present study.

The deuterium tracer study is limited in that it cannot determine the original site of de novo synthesis. It is possible that fatty acids are synthesized in one organ and then circulated through the bloodstream to be taken up by another organ. Therefore, enhanced fatty acid uptake in FR adipocytes remains a possibility. However, the data from the retroperitoneal primary adipocyte cell cultures supports actual differences in de novo synthesis since the cultured adipocytes have been removed from the influence of other organs.

Although the RP fat did not show increased FNS in vivo, the primary adipocyte cell cultures revealed increased de novo lipogenesis after exposure to high glucose medium, which may reflect the cells' response under conditions of nutrient excess. The increased acetyl-CoA enrichment further supports the increased propensity to utilize glucose toward fat storage. These findings support the differences as programmed effects that are manifested in over-nutrition. The differences in de novo synthesis between FR and Controls therefore may not only be a failure to equally suppress de novo lipogenesis under the high fat diet of dam's milk. Alternatively, the differences may be mediated by the increased insulin sensitivity at this age in FR. Further studies are needed to establish the mechanisms by which de novo synthesis is increased.

The increased de novo synthesis in FR adipose tissue during the catch-up growth phase suggests this as the time to intervene to prevent obesity. In adult humans, there is suggestion that de novo synthesis in adipose tissue and the liver may not be a significant contributor to development of obesity [28,29]. However, the role of de novo synthesis may differ in adulthood and infancy. While there is limited tracer-based data showing active substantial de novo synthesis in infancy [30] and ethical considerations in studying infant tissues, de novo synthesis may be a more significant contributor at a young age prior to development of adiposity [10]. Therefore, inhibiting de novo synthesis may potentially be an effective intervention in early life, but not in adulthood.

**Figure 6:**
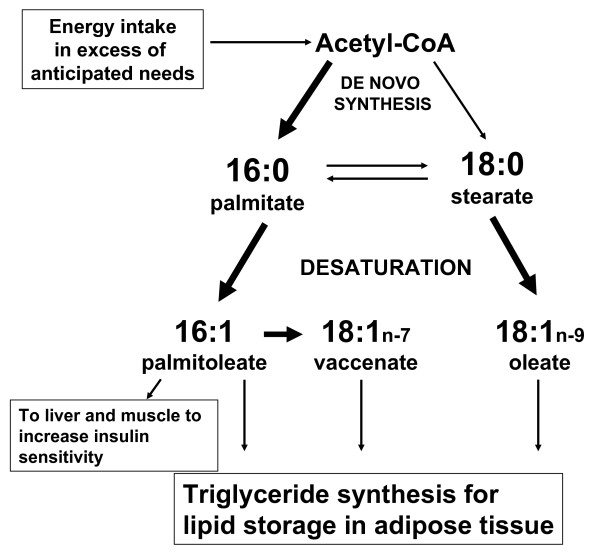
**Summary of alterations in the fatty acid synthesis pathways in FR offspring fat at p21 in vivo**. Arrows with increased boldness indicate increased production. De novo lipogenesis is increased for palmitate in SC fat. The palmitate-to-palmitoleate and stearate-to-oleate pathways are upregulated in SC and RP fat. Vaccenate production is also increased in SC and RP fat through elongation of palmitoleate.

Nutrient restriction prenatally programs the offspring fatty acid metabolism, but the outcome of the programming is dependent upon postnatal nutritional influences. Food-restriction during late gestation leads the offspring's metabolism to adapt to conditions of under-nutrition. During this key developmental phase, the FR offspring is able to reset its balance of energy intake and energy expenditure in anticipation of continuous under-nutrition, optimizing its survival as an adult. This anticipatory physiologic adaptation has been described as the "predictive adaptive response [31]." After birth, however, unexpected exposure to normal nutrition becomes interpreted as over-nutrition, and is therefore a "mismatch [31]" for its metabolism. The FR offspring therefore utilizes the excess energy intake for fat storage, with increased fatty acid de novo synthesis and desaturation in adipose tissue (Figure 6). In human infants born SGA, feeding on nutrient-enriched formula (compared to feeding on control formula) led to increased early weight gain, which was associated with increased fat mass in childhood [32]. Consequently, encouraging early catch-up growth by increasing energy intake leads to over-nutrition for the SGA infant who has adapted to conditions of under-nutrition. Prenatal adaptation, therefore, should definitely be considered in optimizing nutrition for infants born at metabolic risk.

## Conclusion

In summary, increased oleate/stearate desaturation index is present in p1 and p21 FR adipose tissue prior to the onset of obesity. Decreased palmitoleate/palmitate ratio at p1 in contrast to an increased ratio at p21 implicates presence of different factors influencing desaturase activity at the two ages. At p21, there is also increased de novo synthesis in the SC fat in vivo, and in the RP fat in vitro. These differences cannot be explained by de novo synthesis contributions from the dams through nursing. Abnormal lipogenesis is therefore present in FR adipose tissue during the catch-up growth phase. The increased lipogenesis may contribute to ultimate development of adult obesity.

## Competing interests

The authors declare that they have no competing interests.

## Authors' contributions

JKY participated in study design and implementation, data production, data analysis and manuscript preparation. GH participated in the primary adipocyte cell cultures. WNL participated in study design, data analysis and manuscript preparation. MGH participated in manuscript preparation. MD participated in study design, study oversight, data analysis and manuscript preparation. All authors read and approved the final manuscript.
